# Application of image analysis combined with regression models to estimate the reduction of *Escherichia coli* and *Salmonella* spp*.* on vegetable surfaces after washing

**DOI:** 10.1186/s40643-025-00992-4

**Published:** 2026-01-05

**Authors:** Farida Chengsa-ard, Thanakorn Rojanakorn, Pimnibha Hirunsorn, Nattavong Fuangpaiboon, Natthawuddhi Donlao, Yardfon Tanongkankit, Utthapon Issara, Jaspreet Singh, Lovedeep Kaur, Jinhu Tian, Chanthima Phungamngoen

**Affiliations:** 1 Department of Food Technology, Faculty of Technology, Khon Kaen University, Khon Kaen, Thailand; 2 Research Center of Innovative Food Packaging and Biomaterials Unit, Mae Fah Luang University, Chiang Rai, 57100 Thailand; 3 Department of Food Engineering, Faculty of Engineering and Agro-Industry, Maejo University, Chiang Mai, 50290 Thailand; 4 Division of Food Science and Technology Management, Faculty of Science and Technology, Rajamangala University of Technology Thanyaburi, Khlong Luang, Pathum Thani 12110 Thailand; 5 School of Food Technology and Natural Sciences, Massey University, Palmerston North, 4442 New Zealand; 6 Riddet Institute, Massey University, Palmerston North, 4442 New Zealand; 7 College of Biosystems Engineering and Food Science, National-Local Joint Engineering Laboratory of Intelligent Food Technology and Equipment, Zhejiang Key Laboratory for Agro-Food Processing, Integrated Research Base of Southern Fruit and Vegetable Preservation Technology, Zhejiang International Scientific and Technological Cooperation Base of Health Food Manufacturing and Quality Control, Fuli Institute of Food Science, Zhejiang University, Hangzhou, China

**Keywords:** *Escherichia coli*, Image analysis, Organic acids, *Salmonella* spp., Surface roughness

## Abstract

*Escherichia coli*, a bacterium indicating improper hygiene practices during food production, is commonly found in the intestines of humans and animals, while *Salmonella* spp*.* are dangerous bacteria that cause typhoid fever and severe diarrhea. These pathogens have been found in fresh vegetables. This study investigated how the vegetable surface characteristics influenced bacterial adhesion. The reduction of bacteria during the washing process was assessed using different concentrations and types of chemicals. The relationships between variables obtained from image analysis techniques and bacterial adhesion on vegetable surfaces were also evaluated. The most effective way to inhibit bacteria was by washing with 2.0% lactic acid, with bacterial reduction from an initial concentration of 8.74 to 2.92 log CFU/m^2^. Pearson’s correlation with the highest r value was surface area (A) with values ranging from 0.764 to 0.993, followed by surface roughness (R) with values between 0.019 and 0.986, and Fractal dimension (FD) with values between − 0.510 and − 0.992. The correlation between A and the number of bacteria (*E. coli* and *Salmonella*) was the highest, with surface area influencing bacterial adhesion to the vegetable surface. Greater surface roughness was associated with a higher initial bacterial load, making the A value a good predictor of changes in bacteria during washing with organic acids at various concentrations.

**Graphical Abstract:**

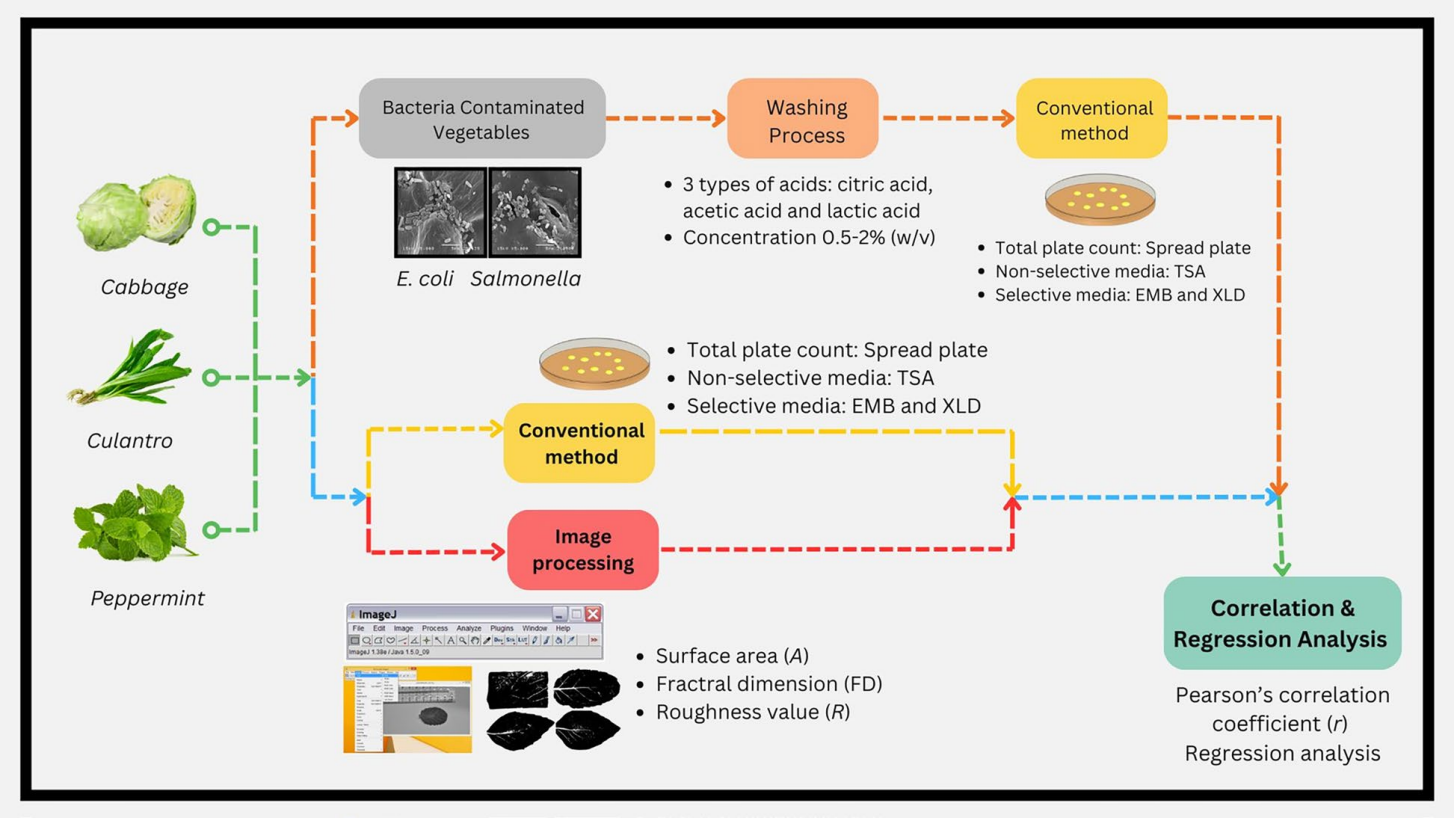

## Introduction

Foodborne pathogens, commonly bacteria, cause gastrointestinal illnesses such as nausea, vomiting, diarrhea, or even neurotoxicity. *Escherichia coli* (*E. coli*), an indicator of improper hygiene during food production, is commonly found in the intestines of humans and animals. It can enter surface rivers through daily wastewater and septic effluents, as well as the use of fecal fertilizers. Pathogenic strains of *E. coli* pose a significant public health risk. *E. coli* spreads to humans through the consumption of contaminated food or drinks, especially uncooked food such as raw meat, raw vegetables, unpasteurized milk and water (Farens and Hovde 2011; Simonne 2023). *Salmonella* is a major foodborne pathogen and a leading cause of gastroenteritis in humans and animals. The transmission of *Salmonella* to humans occurs through the farm-to-table continuum and is commonly linked to the consumption of animal-derived food products. It is a dangerous pathogen that causes typhoid fever and severe diarrhea, leading to symptoms of intestinal inflammation and food poisoning. It is found in the gastrointestinal tract of poultry, as well as in contaminated soil and water. *Salmonella* is frequently detected in various products like chicken, eggs, meat, milk, vegetables, and fruits (Morasi et al. 2022). *E. coli* and *Salmonella* comprise many strains that are found in water and soil, increasing the chances of contamination during the preparation of vegetables. Food poisoning outbreaks from the consumption of fruits and vegetables are often linked to vegetables, especially salad greens, which are handled directly during food preparation, making strict hygiene essential (Erickson et al. 2019). The bacteria are mostly found on contaminated ready-to-eat fruits and vegetables including *Escherichia coli* O157:H7*, Listeria monocytogenes, Shigella, Salmonella* and Hepatitis A virus (Allende et al. 2009; Back et al. 2014; Singh et al. 2002; Wang et al 2019).

The physical properties of food impact food safety. Fruits and vegetables with rough or wrinkled surfaces are more prone to microbial contamination because of their large surface area, which allows bacteria to adhere easily (Phungamngoen and Rittisak 2020). Wang et al. (2009) found that rough or wrinkled fruits and vegetables that underwent cleaning and trimming had significant microbial survival because the uneven surface protected the microbial cells from contact with the cleaning chemicals, thereby impacting food safety. The physical characteristics of food also influence consumer acceptance. High-powered imaging alone cannot quantitatively explain food structural changes, but using quantitative analysis, such as fractal dimension (FD), surface roughness (R), and shrinkage can help explain the structural changes in food materials (Kerdpiboon and Devahastin 2007). Investigating the effect of surface roughness on microbial heat resistance requires the application of image analysis techniques to convert surface roughness into understandable quantitative values. Hawaree et al. (2009) and Chiewchan et al. (2010) developed image analysis techniques to study the surface characteristics of vegetables during the drying process. They described vegetable characteristics using the surface roughness factor (R). Their results showed that the R-value of cabbage increased as drying time lengthened and drying temperature decreased, corresponding to the reduction of moisture content in the samples. Therefore, the R-value can be used as an index to indicate surface changes in vegetables during drying.

Foods with naturally rough or uneven surfaces impact microbial adhesion and the effectiveness of the washing processes to ensure the safety of fresh, ready-to-eat fruits and vegetables. Thus, studying the relationship between vegetable structure and surface characteristics in relation to microbial adhesion and survival after washing is an interesting area of research. One significant factor leading to foodborne illness from fresh vegetables is surface contamination by bacteria (Doyle and Erickson 2008). The most effective way to remove pathogens from food surfaces is through washing. Disinfectants used in industrial food washing processes include acidified sodium chlorite, citric acid (Allende et al. 2009), chlorine, lactic acid (López-Gálvez et al. 2009), and hydrogen peroxide (Back et al. 2014). Washing can reduce pathogen levels below the infectious dose, depending on the effectiveness of the disinfectant. This research focused on *Escherichia coli* and *Salmonella* due to their frequent contamination in vegetables, and investigated how the vegetable surface characteristics influenced bacterial adhesion. The reduction of bacteria during the washing process was assessed using different concentrations and types of chemicals.

## Materials and methods

### Sample preparation

#### Preparation of *Escherichia coli *TISTR 073 and *Salmonella* Typhimurium TISTR 1470

*Escherichia coli* TISTR 073 and *Salmonella* Typhimurium TISTR 1470 (obtained from the culture collection of the Thailand Institute of Scientific and Technological Research; TISTR) were used in this study. These strains were selected because they were originally isolated from human stool samples in Thailand. *E. coli* and *Salmonella* were streaked on slant tubes containing TSA (Tryticase Soy Agar) medium using the streak plate technique. The tubes were incubated at 37 °C for 24 h and then stored at 4 °C as stock cultures. The preparation of *E. coli* and *Salmonella* was adapted from the method of Phungamngoen and Rittisak (2020). The culture was taken from the stock and left at room temperature for 30 min to 1 h. One loop of culture was transferred from the stock tube into 100 mL of TSB (Trypticase Soy Broth) medium in a 250 mL Erlenmeyer flask to provide *E. coli* in the stationary phase. The culture was shaken at 200 rpm and incubated at 37 °C for 18 h, before diluting with 0.1% peptone solution at a ratio of 1 to 100 mL to give a final concentration of 7 log CFU/mL. The cultures of *E. coli* and *Salmonella* were then mixed thoroughly at equal concentrations, resulting in a combined inoculum of both bacteria with a concentration of 8 log CFU/mL.

#### Preparation of vegetable samples

Cabbage, culantro, and peppermint were selected as the study samples. The cabbage and culantro were cut into 2 × 2 cm pieces, and peppermint leaves were selected with uniform size and quality. Before the experiment, the samples were washed with tap water to reduce the quantity of natural microorganisms (normal flora) on the surface. The initial microbial contamination was reduced by approximately 5 log CFU/m^2^ by soaking the vegetables in 70% ethanol (v/v) at a vegetable-to-ethanol ratio of 1:10 (g/mL) for 30 s. The vegetables were soaked in 70% ethanol (v/v) with a ratio of vegetables to ethanol of 1:10 (g/mL) for 30 s, and then rinsed twice with sterile distilled water. The microbial count on the vegetables was measured using TSA (Trypticase Soy Agar), EMB (Eosin Methylene Blue Agar) and XLD (Xylose Lysine Deoxcholate Agar) media. Counting microorganisms on EMB agar and XLD agar, there is no *E. coli* and *Salmonella* on the leaves after washing with 70% ethanol.

#### Inoculation

The prepared vegetable samples were soaked in the inoculum with a vegetable-to-inoculum ratio of 1:20 (g/mL) and then shaken at 120 rpm at room temperature for 1 min. The excess inoculum was discarded, and the vegetables were placed on a sterile cloth and left to air dry in a sterile cabinet for 30 min. The contaminated vegetables were stored in a stomacher bag at 4 °C for 24 h to allow *E. coli* and *Salmonella* to adhere to their surfaces.

### Comparison of chemical types and concentrations for the reduction of *Escherichia coli *and *Salmonella* on the vegetable surfaces during washing

#### Washing process

The contaminated vegetables were washed using citric acid, acetic acid and lactic acid at concentrations of 0.5%, 1.0%, 1.5% and 2.0% v/v. The pKa values of acetic, citric, and lactic acids were determined to be approximately 4.5, 3.2, and 3.9, respectively. The method was adapted from Wang et al. (2015) and Huang and Chen (2011). They were then soaked in 250 mL Erlenmeyer flasks with a ratio of vegetables to chemicals of 1:50 (g/mL) for 3 min. After washing, the remaining amounts of *E. coli* and *Salmonella* on the vegetable surfaces were evaluated.

#### Enumeration of microorganisms on the vegetable surfaces

The washed vegetable samples were mixed with peptone solution at a ratio of 5:50 (g/mL) in stomacher bags, and then homogenized at 230 rpm for 2 min. The bacterial count was determined using the spread plate method using TSA as a complete medium and EMB and XLD as selective media. The remaining *E. coli* on the vegetable surfaces formed black colonies with a metallic sheen on EMB, while the remaining *Salmonella* on the vegetable surfaces formed black colonies on XLD with a pink or red background. The results were expressed in log CFU/g and converted to log CFU/m^2^ using the formula:

1 $$ {\text{log}} \,{\text{CFU}}/{\text{m}}^{{2}} = \frac{{{\text{Number of colonies counted}} \times {\text{dilution factor}} \times 10}}{{{\text{leaf area value}} }} $$

### Surface analysis of vegetables using image analysis techniques

#### Surface area measurement using the weighing method

Twenty fresh leaves from each sample were used to measure the surface area to determine the accuracy of the image analysis technique.

The surface area was calculated using proportional reasoning as:

2 $$ {\text{Leaf area}} = \frac{{{1} \times {\text{A}}}}{{\text{B}}} $$

The leaf outline was drawn on a sheet of A4 paper and cut out. The cutout paper was then weighed (A). The weight of the pieces was used to calculate the average weight (B).

#### Surface area (A) and fractal dimension (FD) measurements using image analysis

**Fig. 1:**
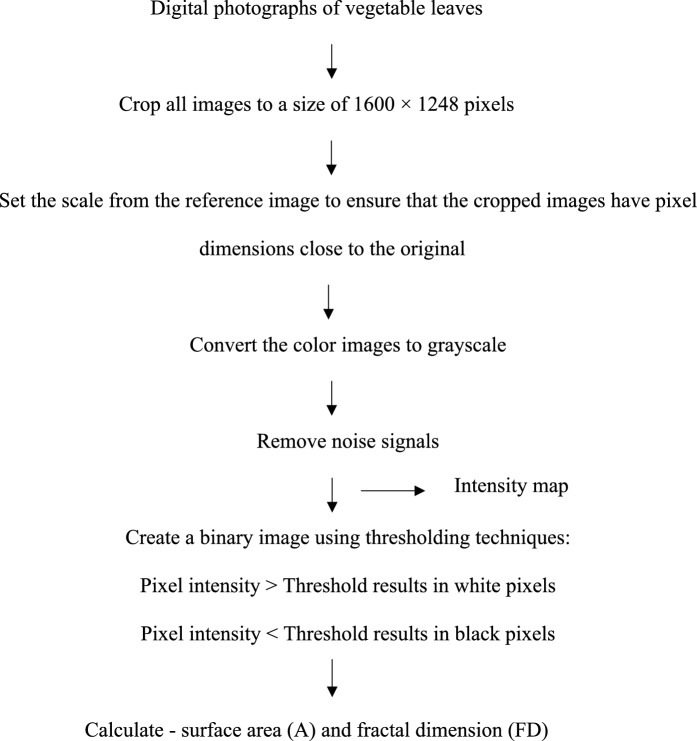
Schematic to evaluate the vegetable surface area (A) and fractal dimension (FD)

Twenty fresh leaves of each sample were photographed using a digital camera (Olympus m.zuiko digital 14–42 mm lens). The samples were placed in a controlled-light photo box, and both the front and back sides of the leaves were photographed. The camera lens was zoomed to 40 mm for cilantro, cabbage, and mint leaves, with 15 cm between the camera and each sample. The captured images were then analyzed using ImageJ software (version Fiji 1.52p) to determine the surface area (A), surface roughness (R), and fractal dimension (FD) of each type of vegetable, following the method of Phungamngoen and Rittisak (2020). The images used to measure length or area had varying pixel densities or resolution (sharpness). ImageJ software measures dimensions in pixels, the fundamental unit of an image, instead of standard-length units. An image resolution of 1600 × 1248 pixels means that the image is composed of 1600 pixels in length and 1200 pixels in width. Before measuring the actual length or area on the image, the pixel resolution must be calibrated into standard units for accurate measurement of the real dimensions of objects in the image, as shown in Fig. 1.

#### Calculation of the surface roughness factor (R)

**Fig. 2:**
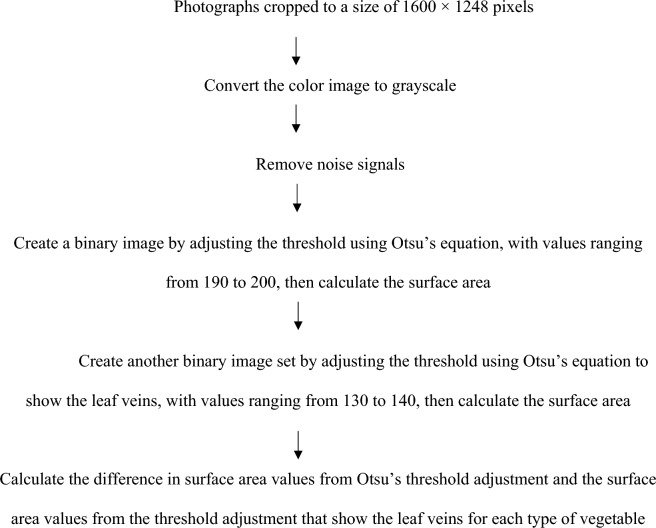
Steps for calculating the surface roughness factor (R)

Photographs of the vegetable leaves from each sample were used to calculate the surface area by adjusting the threshold using Otsu’s method, resulting in a completely black image. The surface area was calculated from the threshold adjustment that showed the leaf veins of each type of vegetable. The difference between the surface area values from Otsu’s threshold adjustment and the surface area values from the threshold adjustment showing the leaf veins was used as the surface roughness factor for each type of vegetable. The procedure for calculating the surface roughness factor (R) was referenced from the research of Phungamngoen and Rittisak (2020), as shown in Fig. 2.

### Micrograph of fresh cabbage surface by scanning electron microscope (SEM)

The appearance of different fresh cabbage surface was evaluated follow methods of Phungamngoen et al. (2011) with some modifications. Attachment of cocktail bacteria (*E. coli* and *Salmonella*) on fresh cabbage surface were observed. The surface of samples was coated with platinum for SEM imaging quality. The samples were put on to a (FE-SEM, Zeiss, Auriga) scanning electron microscope and central areas were photographed at magnifications at ×1000 and ×5000.

### Statistical analysis

This research was divided into two parts. The first part consisted of image analysis using a completely randomized design (CRD). The factors included the type of vegetable, with surface roughness, surface area, and fractal dimension from the image analysis technique serving as response variables. The second part examined the adhesion of *E. coli* and *Salmonella* on the vegetable surfaces using a 3 × 3 × 4 CRD. The first factor was the type of vegetable, divided into three levels: cabbage, culantro and peppermint. The second factor was the type of antimicrobial chemical, divided into three levels: citric acid, acetic acid, and lactic acid. Finally, the third factor was the concentration levels of the antimicrobial chemicals, divided into four levels: 0.5%, 1.0%, 1.5%, and 2.0%. The statistical differences in the data were analyzed using SPSS (version 16.0) at a confidence level of 95%, with three replications for each experiment, and the results were presented as the mean and standard deviation.

## Results and discussion

### Surface characteristics of different vegetables

Table 1 shows the color images of the three different vegetables. A clear cell periphery and network of venation were visible on the surfaces of the fresh vegetables. Different types of vegetables showed different patterns of venation. The upper side of the leaves showed the network venation more clearly than the lower leaf side, concurring with Phungamngoen et al. (2011). In cabbage, the upper and lower sides showed similar smooth but wavy surface roughness, with the leaf curving inward and tightly overlapping (Wang et al. 2009). By contrast, the upper and lower sides of the culantro and peppermint leaves differed significantly in roughness, with veins on the upper side of the leaf smoother than those on the lower side. Rezai et al. (2018) reported that the upper side of the leaf was exposed to sunlight, used for chlorophyll synthesis, and tended to be darker green with a smoother surface compared to the back of the leaf, where the veins were more prominent, aligning with observations made by sight and touch (Phungamngoen and Rittisak 2020).

**Table 1:**
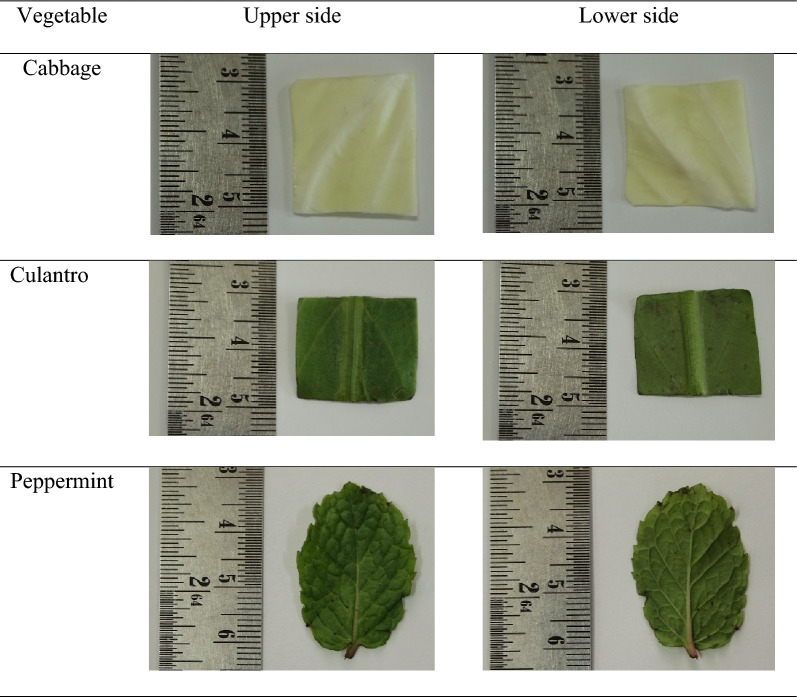
Colored images of the three vegetables

The surface roughness analysis of each vegetable leaf was performed by capturing leaf images and calculating the surface area using Otsu’s threshold method. The resulting binary black and white images were used to determine the surface area from the threshold adjustment to determine the surface roughness factor (R) and fractal dimension (FD), as shown in Table 2. Results showed that the actual surface areas calculated using the weighing method and the surface areas from image analysis (A) for the three types of vegetables were not statistically different. Image analysis was used to calculate the surface area of the vegetables. Peppermint showed the highest surface roughness and surface area resulting from the most prominent vein ridges, followed by culantro and cabbage.

**Table 2:** Surface area from the weighing method and parameters from image analysis techniques of each vegetable leaf

Vegetable type	Conventional method	Parameters from image analysis techniques		
	Surface area (cm^2^)	Surface area (A)	Fractal dimension (FD)	Roughness factor (R)
Cabbage	4.14 ± 0.20^bA^	4.13 ± 0.11^bA^	1.63 ± 0.05^aB^	0.27 ± 0.20^bC^
Culantro	4.20 ± 0.12^bA^	4.16 ± 0.14^bA^	1.65 ± 0.08^aB^	0.29 ± 0.20^bC^
Peppermint	5.75 ± 0.48^aA^	5.71 ± 1.10^aA^	1.12 ± 0.02^bB^	0.41 ± 0.32^aC^

^a^^−^^c^indicates significant differences in vertical letters at a 95% confidence level

^A^^−^^C^indicates significant differences in horizontal letters at a 95% confidence level

The fractal dimension (FD) values of each vegetable leaf were analyzed using the ImageJ program. A value close to 2 indicates that the leaf image has low roughness, while values further from 2 indicate greater roughness (Valous et al. 2009; Mirzabe et al. 2017; Mohammadi et al. 2015). Peppermint had the lowest FD, indicating a surface showing the highest irregularity. The FD represents the two-dimensional or three-dimensional area of the surface, consisting of three characteristics: color, shape, and texture. Color is a prominent feature that plays a significant role in image retrieval systems; it is easily distinguishable and helps differentiate objects within an image. Shape describes the form and size of objects within an image, allowing for the separation of objects with different shapes. Texture refers to the coarseness, fineness, or complexity of objects in an image. Different objects may have varying textures, and analyzing these textures helps to distinguish between objects. The fractal dimensions of the vegetables concurred with Quevedo and Aguilera (2004). They applied fractal image texture analysis to describe the texture and microstructure of various foods such as pumpkin, potato, carrot, apple, and banana. Higher values of fractal dimension represent more complex or rougher surfaces. The fractal dimension correlated well with the visual characteristics and roughness of standard material (sandpaper). As mentioned earlier, FD could be used as an indicator to monitor the surface characteristics of vegetables.

### Attachment of *Escherichia coli* and *Salmonella* spp. on the vegetable surfaces

**Fig. 3:**
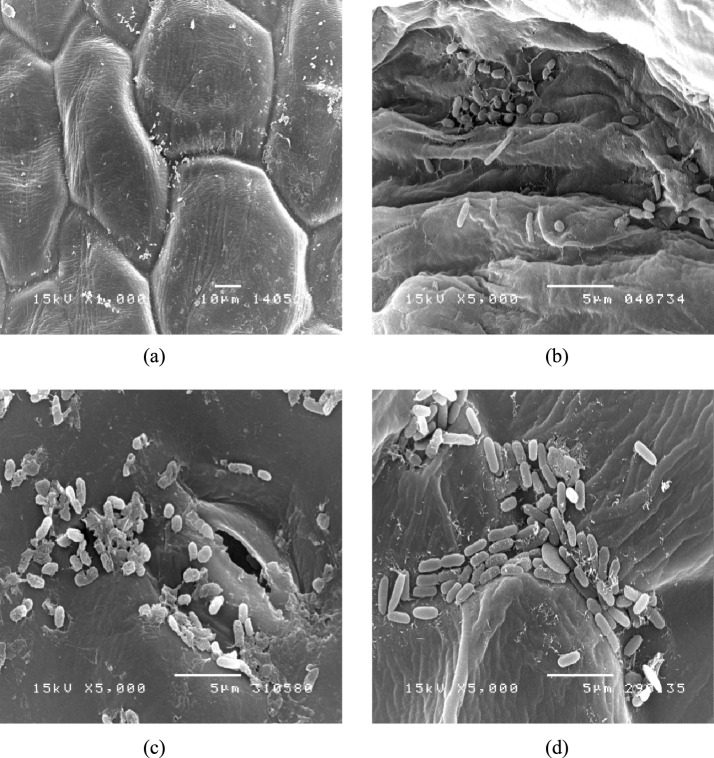
Attachment of *cocktail bacteria (E. coli* and *Salmonella)* on fresh cabbage surface at different areas: **a** leaf cells; **b** leaf edge; **c** stomata; **d** leaf veins

Table 3 shows the initial bacterial adhesion on different vegetable surfaces. The microorganisms were counted on EMB agar and XLD agar, with *E. coli* and *Salmonella* not found on the leaves after washing with 70% ethanol. The initial microbial contamination was reduced by 5 log CFU/m^2^ compared to the initial contamination levels. The surfaces of the leafy vegetables showed rough venation patterns that promoted the entrapment and attachment of bacteria. *E coli*, *Salmonella*, and the cocktail bacteria were higher on peppermint than cabbage and culantro because the bacterial cells were distributed on the smooth surface and preferably attached around the stomata and rough surfaces, such as veins of leafy vegetables and the netted rind surfaces of some fruits (Ells and Hansen 2006; Hawaree et al. 2009). The SEM micrographs showed the population of cocktail bacteria attachment on the cabbage surface (Fig. 3). The results concurred with Wang et al. (2009), who studied the effect of surface roughness on the retention and removal of *Escherichia coli* O157:H7 on the surfaces of golden delicious apples, navel oranges, avocadoes, and cantaloupes. The bacterial adhesion rate increased with higher surface roughness due to the increase in surface area. Moreover, surface roughness exerted a stronger effect on bacterial removal during washing than surface hydrophobicity, likely due to the larger available surface area for bacterial attachment. Based on microstructural characteristics, three types of fruit surfaces can be distinguished: (1) smooth surfaces with few irregularities (e.g., apples), where attached bacteria are readily exposed to shear forces from washing; (2) moderately rough surfaces with shallow valleys (e.g., oranges and avocados), where bacteria are partially protected; and (3) highly rough surfaces with deep cavities and wide grooves (e.g., cantaloupes), where bacteria are well shielded from mechanical forces during washing.

**Table 3:** Initial bacterial adhesion on each vegetable surface

Vegetable type	Initial microbial count (log CFU/m^2^)		
	*Escherichia coli*	*Salmonella* spp.	Cocktail bacteria
Cabbage	7.71 ± 0.08^bB^	7.52 ± 0.09^bC^	8.17 ± 0.08^bA^
Culantro	7.41 ± 0.08^cB^	7.11 ± 1.00^cC^	7.96 ± 0.07^aC^
Peppermint	8.74 ± 0.17^aA^	8.08 ± 0.08^aB^	8.83 ± 0.09^aA^

^a^^−^^c^indicates significant differences in vertical letters at a 95% confidence level

^A^^−^^C^indicates significant differences in horizontal letters at a 95% confidence level

Peppermint had the highest microbial count after contamination due to its rough surface, while cabbage had fewer remaining microorganisms despite its low surface roughness. The cabbage was cut into 2 × 2 cm pieces before contamination and the cuts and bruising made it easier for the microorganisms to attach. These results concurred with Kolattukudy (1981) and Leide et al. (2012), who found that wounds made vegetables more susceptible to microbial contamination. Culantro had the fewest attached microorganisms, with the lowest surface roughness and a smoother and thinner structure than cabbage. Culantro was only cut on two sides, resulting in fewer wounds and reduced microbial attachment.

### The effect of different organic acid concentrations on reducing bacterial populations after the washing process

**Fig. 4:**
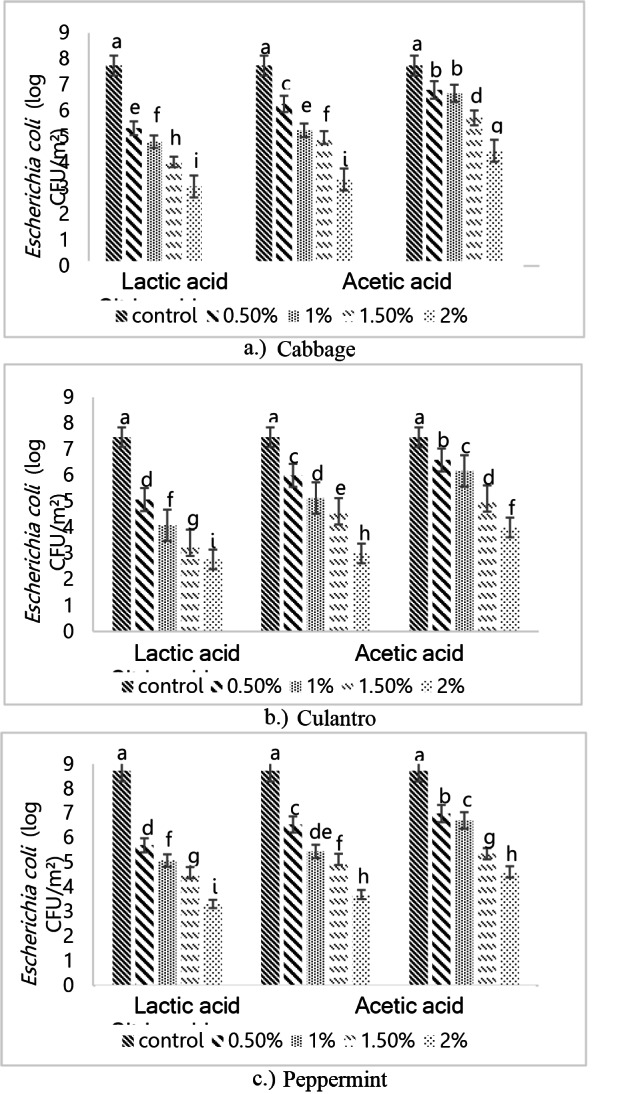
*Escherichia coli* attachment on the surfaces of the three vegetables after washing with different organic acids

Figure 4 shows *E. coli* attachment on the surfaces of the three vegetables after washing for 3 min with different organic acids. The initial microbial contamination was 7.47–8.73 log CFU/m^2^. After washing with 2% citric acid, the microbial count reduced to 4.0–4.6 log CFU/m^2^, concurring with Huang and Chen (2011). They reported that 2% citric acid reduced *E. coli* counts on peppermint leaves at 5.43 log CFU/g by 1.16 log CFU/g. Citric acid inhibits microbial growth by impacting ATP synthesis during electron transport or by inhibiting the movement of metabolites within the cell (Nielsen and Arneborg 2007). Van Immerseel et al. (2007) also reported that organic acids penetrate microbial cells, increasing the internal acidity and disrupting DNA synthesis, leading to bacterial cell death. Acetic acid washing reduced the microbial count on all the three vegetables to 3.0–3.70 log CFU/m^2^ from an initial 7.47–8.73 log CFU/m^2^, with significant statistical differences (*P* < 0.05). Post-wash, slight browning of the leaves was observed, consistent with Sengun and Karapinar (2004). They found that 2% acetic acid reduced *E. coli* counts from 6.41 log CFU/g to 1.57–3.58 log CFU/g. Acetic acid disrupted the cell membrane, reducing the pH within microbial cells and impacting metabolic processes. Lactic acid at 2% reduced the microbial counts on the three vegetables to 2.77–3.32 log CFU/m^2^. Wang et al. (2019) reported that lactic acid caused protein leakage from cells. Structural damage to the bacterial cells was observed, including severe membrane damage that impaired cell functions.

**Fig. 5:**
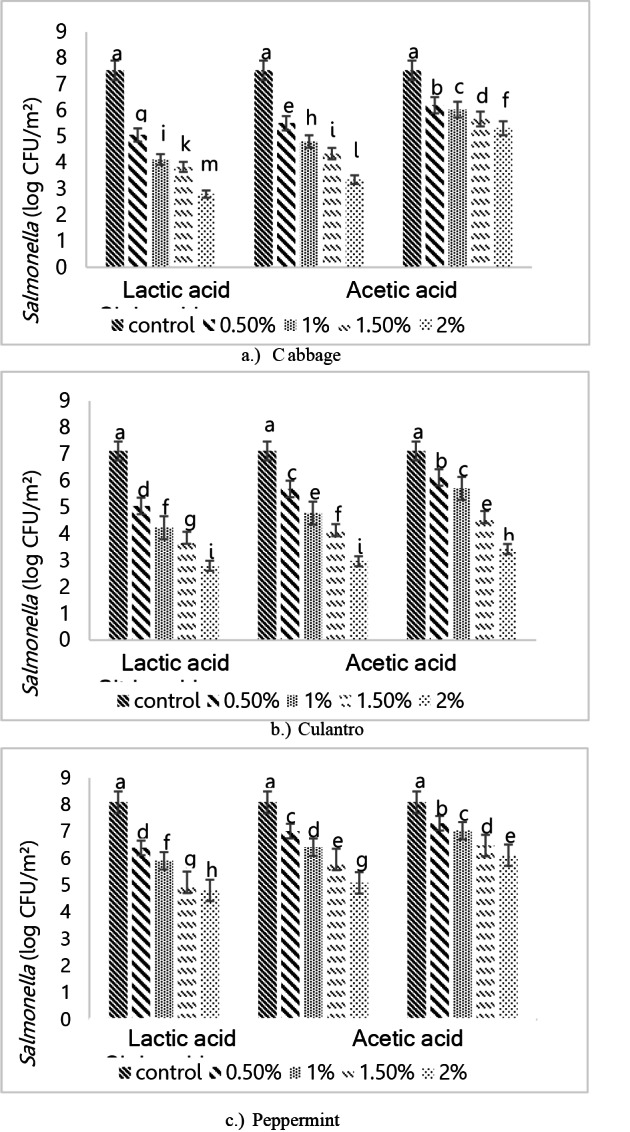
*Salmonella* spp. attachment on the surfaces of the three vegetables after washing with different organic acids

Figure 5 shows *Salmonella* attachment on the surfaces of the three vegetables after washing with different organic acids. Lactic acid at 2% was the most effective for reducing microbial counts on all three vegetables, consistent with the results shown in Fig. 4. *Salmonella* and *E. coli* are both gram-negative Enterobacter bacteria. The washing effects on *Salmonella* showed that more microorganisms remained on the vegetable surface compared to *E. coli*. This was due to *Salmonella* flagella (Wang et al. 2015; Kwon et al. 2019), which helped the bacteria to adhere better than *E. coli* by enabling microbial movement to optimize survival and food location, allowing the bacteria to move away from harmful substances or environments.

**Fig. 6:**
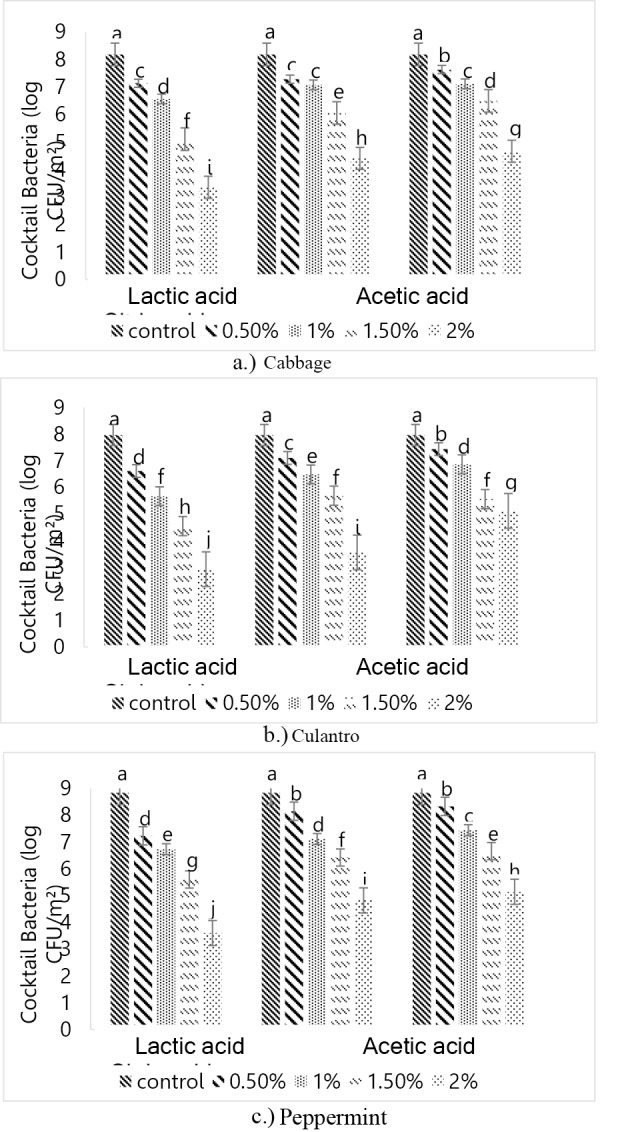
Cocktail bacteria attachment on the surfaces of the three vegetables after washing with different organic acids

*E. coli* and *Salmonella* were combined into a “cocktail bacteria” mix and washed with different acids, as shown in Fig. 6. Acetic acid was more effective than citric acid at room temperature. This finding aligned with Al-Nabulsi et al. (2014). They showed that acetic and citric acids reduced *S*. Typhimurium in tabbouleh salad. Citric acid at concentrations of 0.4% and 0.8% reduced *S*. Typhimurium, *E. coli* O157:H7, and *S. aureus* by 1.5 log CFU/g. The antimicrobial activity of organic acids depends on the concentration and pKa value, which makes them more effective under acidic conditions. Organic acids penetrate bacterial membranes and lower the internal pH of the cells, further disrupting cell processes. Other factors, such as membrane disruption and ion accumulation, may also contribute to the antimicrobial activity of organic acids (Doores 2005; FDA 2014). The stronger inhibition of acetic acid compared to citric acid was explained by the greater ability of the former to penetrate bacterial membranes due to its lower molecular weight (faster diffusion), higher pKa, and greater hydrophobicity (Fernández et al. 2009). Lactic acid at 2% was the most effective at inhibiting microbial growth on the three vegetables. This finding concurred with Kwon et al. (2019). They reported that 2% lactic acid inhibited *Escherichia coli* O157:H7, *Salmonella* Typhimurium, and *Listeria monocytogenes* on the surface of cantaloupe without compromising the quality of the fruit.

### The relationship between variables obtained from image analysis techniques and the adhesion ability of *Escherichia coli* and *Salmonella* spp. on vegetable surfaces

The results of Pearson’s correlation between the variables obtained from image analysis techniques, and the ability of *E. coli* and *Salmonella* to adhere to the surfaces of the vegetables are shown in Table 4. There was a significant correlation between the initial contamination levels on the three vegetables and the surface area (A), surface roughness factor (R), and FD values. A correlation coefficient (r) close to 1 or − 1 indicates a strong correlation. A value of 1 represents a perfect positive correlation, while a value near 0 indicates little to no correlation. If r = 0, there is no linear correlation (Kozak and Bornmann 2012). A good correlation was found, with a high Pearson’s correlation indicating that a rough surface area significantly affected *Salmonella* concentrations on the vegetable surfaces, with a linear relationship observed. The variable with the highest correlation (r) was surface area (A), with values ranging from 0.596 to 0.993, followed by FD with values from − 0.510 to − 0.992 and the surface roughness factor (R) with values from 0.019 to 0.986. A and R increased with increasing bacteria on the surface of the vegetables. The bacterial adhesion rate increased with higher surface roughness, with the surface topographical features of vegetables assisting the attachment of bacteria. The bacterial cells were distributed on the smooth surface and preferentially attached to rough surfaces such as the stomata of leafy vegetables and the netted rind surfaces of some vegetables (Chiewchan and Morakotjinda 2009; Phungamngoen et al. 2011) due to the increase in surface area for bacterial adhesion. By contrast, FD decreased with increasing bacteria on the vegetable surfaces. An increase in cellular contraction, shown by the clear vein patterns, gave higher A, R, and lower FD values. The correlation between surface area (A) and *Salmonella* was the highest compared to R and FD. The surface area (A) influenced the adhesion of *Salmonella* on the vegetable surfaces and was a good predictor for bacterial adhesion. This study investigated the impact of food surface characteristics on the reduction of bacteria during the washing process with organic acids. The A value was selected to predict the bacterial count reduction during the washing process, with the studied factors being the type of organic acid, the concentration of acid, and the A value. The relationships between the number of bacteria and these three factors with *R*^2^ and adjusted *R*^2^ were as follows:

3 $$ \begin{aligned}& Salmonella \, ({\text{log CFU}}/{\text{m}}^{{2}} ) = { 4}.{81 }{-}{ 2}.0{\text{3x }} \\&\quad + \, 0.{6}0{\text{y }} + \, 0.{\text{34z}};R^{{2}} = \, 0.{917},\\&\quad{\text{ adjusted }}R^{{2}} = \, 0.{917} \end{aligned}$$

4 $$ \begin{aligned}& E. \, coli \, ({\text{log CFU}}/{\text{m}}^{{2}} ) = { 5}.{79 }-{ 1}.{\text{99x }} \\&\quad + \, 0.{\text{45y }} + \, 0.{\text{39z}};R^{{2}} = \, 0.{924},\\&\quad{\text{ adjusted }}R^{{2}} = \, 0.{919} \end{aligned}$$

5 $$ \begin{aligned}& {\text{Cocktail bacteria }}({\text{log CFU}}/{\text{m}}^{{2}} ) = { 4}.{81 }-{ 2}.0{\text{3x }} \\&\quad+ \, 0.{6}0{\text{y }} + \, 0.{\text{34z}};R^{{2}} = \, 0.{896},\\&\quad{\text{ adjusted }}R^{{2}} = \, 0.{885}\end{aligned} $$

where x = concentration of acid (% v/v). y = A value (surface area of leafy vegetable from image analysis technique; m^2^). z = types of acid (lactic acid = 4, acetic acid = 2 and citric acid = 3).

**Table 4:** Pearson’s correlation between variables obtained from image analysis techniques and the ability of *Escherichia coli* and *Salmonella* to adhere to the vegetable surface

Vegetable type	A		FD		R	
	r	P	r	P	r	P
a. *Salmonella*						
Cabbage	0.912	0.023	− 0.598	0.119	0.052	0.736
Culantro	0.839	0.045	− 0.971	0.036	0.748	0.022
Peppermint	0.917	0.011	− 0.991	0.041	0.019	0.582
b. *Escherichia coli*						
Cabbage	0.596	0.136	− 0.737	0.023	0.986	0.027
Culantro	0.764	0.013	− 0.510	0.093	0.414	0.245
Peppermint	0.993	0.034	− 0.992	0.014	0.516	0.018

FD, A, and R represent fractal dimension, surface area, and surface roughness, respectively

## Conclusions

This study compared the effects of chemicals to reduce *E. coli* and *Salmonella* on the surface of vegetables during the washing process. The impacts of the structural characteristics of the vegetable surfaces on the adhesion ability of *E. coli* and *Salmonella* were investigated, together with the optimal concentration and type of chemicals. The relationship between the parameters obtained from image analysis techniques and the adhesion ability of *E. coli* and *Salmonella* on the surface of vegetables was also analyzed. Peppermint leaf had the highest surface area (A) and surface roughness factor (R), with the lowest fractal dimension (FD). This resulted in a greater number of bacteria adhering to the surfaces of mint leaves compared to the other two vegetable types studied. As the concentration of acid increased, the number of microorganisms significantly decreased. Lactic acid was the most effective organic acid in reducing the number of microorganisms. Based on Pearson’s correlation analysis, the variable with the highest r value was A, ranging from 0.596 to 0.993. The second highest was R, ranging from 0.019 to 0.986, and the FD value ranged from − 0.510 to − 0.992. The correlation between A and the number of *E. coli* and *Salmonella* was the highest, as the surface area significantly impacted the adhesion of both bacteria on the surfaces of the vegetables. Therefore, A could be used as a variable for image analysis to predict changes in bacteria during washing with organic acids at various concentrations. The A value was used to create a regression equation to predict the reduction in bacterial count during washing. However, the large number of microorganisms used in this study (7–8 log CFU/mL) made it impossible to completely remove the microorganisms from the vegetables surfaces. Therefore, further studies are required to determine the effect of inoculum size for microorganism contamination on vegetable surfaces.

## Data Availability

All data generated or analyzed during this study are included in this published article.
